# Efficacy of biological response modifier lentinan with chemotherapy for advanced cancer: a meta‐analysis

**DOI:** 10.1002/cam4.1156

**Published:** 2017-09-21

**Authors:** Hui Wang, Yong Cai, Yue Zheng, Qixuan Bai, Dongling Xie, Jiufei Yu

**Affiliations:** ^1^ Department of Gastroenterology Civil Aviation General Hospital Beijing 100123 China

**Keywords:** Advanced cancer, gastrointestinal cancer, lentinan, lung cancer, meta‐analysis

## Abstract

Lentinan is a common biological response modifier. This study was sought to evaluate the efficacy of adjuvant lentinan combined with chemotherapy for advanced cancer. A meta‐analysis of published prospective controlled trials investigating the effects of lentinan for kinds of advanced cancer was performed. Sensitivity analysis, inverted funnel plots, and trial sequence analysis were conducted to explore the reliability and stability of results. Seventeen clinical studies were identified containing 1423 patients. Twelve trials included gastrointestinal cancer (GIC), three trials included lung cancer (LC), and two trials included the two cancers. There was a increase in survival rate in 1 year (risk ratios [RR], 1.46, *P *=* *0.001) and overall response rate including both complete and partial response (RR, 1.28, *P *=* *0.005). There was also a reduction in progressive disease (RR, 0.57, *P *=* *0.0005), nonsevere adverse events (RR, 0.88, *P *=* *0.004), and severe adverse events (RR, 0.73, *P *=* *0.007). Similar results were shown in the two subgroups of GIC and LC. Limited trials reported the data of median overall survival and time to treatment failure, and the data were insufficient for quantitative analysis, and no significant difference were found in 2‐year survival rate. Adjuvant lentinan used with chemotherapy achieved improvements in 1‐year survival rate, response rate, and adverse events in advanced cancer. The effect seemed to be similar irrespective of cancer type. However, its sustained efficacy on survival was still unclear.

## Introduction

Biological response modifiers (BRMs) were generally defined as agents or approaches modify the relationship across tumor/other diseases, treatment, and host by modifying the host's biological response with resultant therapeutic effects 1. Always, BRMs may have one of modifying abilities such as increasing the host's defense, tumor identification, tumor maturation, antitumor response, or tolerance of cytotoxicity by chemotherapy 1, 2. Among kinds of BRMs, lentinan as a fungal polysaccharide agent is approved and widely used to adjuvant therapy for advanced or recurrent cancer in Asian including China and Japan for more than two decades.

Purified from Shiitake mushroom, a tradition food with medical effects, active ingredient of lentinan is *β*‐1, 3‐D‐glucan polymer with *β*‐1, 6 or *β*‐1, 4 branches 2. Series of bioactive research, animal studies, and clinical trials are designed and performed to explore the efficacy of additional lentinan combined with first‐line chemotherapy for kinds of diseases and malignant tumors for a long time 3, 4. A previous individual analysis yielding 650 patients diagnosed as unresectable and recurrent gastric cancer (GC) from year of 1979 to 1989 in Japan focused on overall survival (OS) and safety showed a very promising result 5. The participants received chemotherapy regimens of mitomycin C and 5‐fluorinated pyrimidine (5‐FU)/tegafur, and lentinan significantly improved medial survival time by 25 days without any severe adverse events, and adjusted hazard ratio for OS was 0.76 with a *P* value of 0.002. Lots of clinical trials adopted multiple outcome measures were completed after that meta‐analysis, however, some inconsistent results were presented 6. As a nonspecific BRMs, some researchers also attempted to apply additional lentinan as a chemo‐immunotherapy in kinds of other cancers such as lung cancer (LC) and metastatic liver cancer 7.

Despite the widely use and promising results, there has yet to be a comprehensive review of all available evidence with accumulative data to evaluate the application of lentinan in multiple outcome measures. We have systematically evaluated the clinical outcomes of adjuvant lentinan combined with chemotherapy for all kinds of cancers by gathering published prospective clinical controlled studies in this meta‐analysis.

## Methods

This study was reported mainly according to the items of PRISMA guideline. Study selection and data abstraction were both completed by two independent reviewers, and the results were confirmed and determined by a third one only when disagreements existed.

### Online literature search

Eligible literature was identified by searching abstracts in online databases including PubMed, EMBASE, the Cochrane Library, Google Scholar, and China Knowledge Resource Integrated Database (CNKI). Free‐text terms were adopted and improved by primary reading relevant studies and reviews, and different search strategies were tried in databases to determine an optimal one as follows: (“lentinula edodes mycelia extract” OR “lentinus edodes” OR lentinan OR LTN OR “shiitake mushroom” OR LEM OR “beta‐1,3‐glucan”) AND (carcinoma OR cancer OR tumor OR carcinoma OR malignant OR advanced OR stomach OR gastric OR liver OR hepatic OR hepatocellular OR pancreas OR pancreatic OR colon OR colorectal) AND (randomized OR randomized OR randomize OR randomize OR placebo OR random OR randomly OR control). Published language was limited to English and Chinese, and published time was limited to January 12, 2017 in each database. Besides, related articles in databases and references of finally identified papers were also checked backwards.

### Inclusion and exclusion process

Only studies designed as prospective controlled clinical studies and investigated the efficacy of adjuvant lentinan for advanced cancer were considered. During this selection process, abstracts of the searched literature were screened and handled according to inclusion criteria, and full‐texts were further read to ensure the suitability of potential abstracts. Detailed inclusion criteria were listed as following: (1) participants were patients diagnosed and histologically proven as advanced cancer including gastrointestinal cancer (GIC), GC, esophagus cancer, hepatocellular carcinoma (HCC) or LC; (2) interventions included chemotherapy drugs such as adriamycin, cyclophosphamide, cisplatin, daunorubicin, fluorouracil and so on based on different kinds of cancers. Compared with patients only received chemotherapy in the control group, additional lentinan was added to patients in the treatment group (lentinan group). To ensure its stability and accuracy of active ingredient, lentinan should be prepared as a medicine by intravenously injection or local perfusion, other than rough tablet preparations by orally taking. Specific dose and course of lentinan were not limited only when they were comparable between the groups in each separate trial; (3) outcome measures should include at least one kind of efficacy or safety index that presented as mean and standard deviation or 95% confidence interval (CI). Not relevant studies, case series, animal studies, retrospective clinical case–controls, and reviews were excluded.

### Data abstraction and outcome measures

Baseline characteristics and outcome measures were both abstracted from full‐texts of the included trials. Baseline characteristics included first author, published year, case number, age, sex, diagnosis, chemotherapy regimen, dose and course of lentinan, and follow‐up period.

Primary outcome measures were survival rate and survival time. Secondary outcome measures were time to treatment failure, overall response rate, complete response and partial response, progressive disease, and adverse events. Response to treatment was mainly judged by computed tomography scan according to RECIST version 1.0 to assess the size of measurable lesions every month 8. Adverse events were mostly graded mainly according to National Cancer Institute Common Terminology Criteria for Adverse Events. Adverse event graded more than 2 was regarded to be a severe one, otherwise it was regarded as a mild or moderate one.

### Risk of bias assessment

The quality of the included studies was systematically evaluated by the tool of risk of bias from the Cochrane Library 9. Potential high, low, or unclear risk of bias from process of selection, performance, detection, attrition, reporting, and others were all covered. A total of six detailed items including random sequence, allocation concealment, blinding of patients and outcomes assessments, incomplete outcome data, selective reporting, and others bias were designed for each study. Proper steps were took to prevent or avoid such bias indicated low risk, whereas improper or no steps were stated to took indicated high risk, and exceptions were unclear risk.

### Statistical analysis

Quantitative analysis was performed using Review Manager (RevMan) 5.3 software (the Cochrane Collaboration, Denmark) which was provided by the Cochrane Collaboration. Interstudy heterogeneity was tested using the Cochran *Q* statistic (chi‐square value), with the significance level set at a *P* value less than 0.10 and was quantified by using the *I* ^2^ statistic, where a value of 50% or greater indicates substantial heterogeneity. The occurrence of heterogeneity across the trials for analyzing each outcome measure determined the combined model. When I^2^ ranged from 0% to 50%, a fix‐effects model was used, otherwise a random‐effects model was used when *I* ^2^ ranged from 51% to 100%. Combined effect estimates were presented in risk ratios (RR) for dichotomous outcomes and in standard mean division (SMD) for continuous outcomes with both of their corresponding 95% CI and *P* value. Difference together with a *P* value less than 0.05 was considered substantial significant. Sensitivity analysis was performed though omitting each study in steps for the source of heterogeneity, and after omitting one or more studies which led to a significant *I* ^2^ value decrease the changing trends of combined effect estimates was compared to test the stability of the results. Subgroup analysis though stratifying baseline characteristics (different kinds of cancers including GIC, LC, and others) were conducted to explore the difference across kinds of cancers as well as to lower the influence from kinds of corresponding first‐line chemotherapy regimens; and subgroup analysis was conducted though distinguishing severity and specific diagnosis for adverse events. Publication bias was explored by inverted funnel plots based on their asymmetry, and as well as the statistic method of Egger's test and Begg's test with quantitative analysis results using Stata 12.0 software (StataCorp., College Station, Texas).

Besides, to avoid a false‐positive result, trial sequence analysis (TSA) was conducted by TSA software (version 0.9.5.5 Beta, Centre for Clinical Intervention Research, Copenhagen, Denmark) 10. To avoid type I errors, group sequential monitoring boundaries are applied to decide whether a trial could be terminated early because of a sufficiently small *P*‐value, that is if the cumulative *Z*‐curve crosses the monitoring boundaries. Also, TSA would estimate the required sample size based on current combined data retrieved from each separate trial. Descriptive analysis was performed when there was limited number of trials regards on some specific items which may be useful for further research.

## Results

### Summary of the selected trials

**Figure 1:**
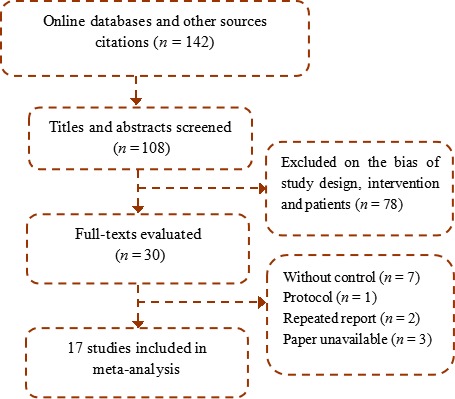
Trial selection.

Online search yield a total of 142 reference with 34 duplicates. Of these, 17 clinical studies (fifteen random trials and two controlled studies) met the inclusion criteria and were finally included after reading their full‐texts 11, 12, 13, 14, 15, 16, 17, 18, 19, 20, 21, 22, 23, 24, 25, 26, 27, as shown in Figure 1. The meta‐analysis encompassed 1423 patients from China and Japan between 1992 and 2016. There were 718 cases in the lentinan group and 705 cases in the control group, with ranged case number from 15 to 149 in the arms of each trial. Average age and sex were listed in Table 1, which were stated to be comparable in each included trial. All the patients had malignant solid tumors, which were recurrent or lost the chance to operation, including advanced GC in seven trials, advanced LC in three trials, HCC in two trials, and esophagus cancer in one trial; whereas GC and LC patients were analyzed together in two trials 13, 25 and GIC patients were gathered together in the other two trials 14, 22.

**Table 1:** Basic information of the included studies

Study	Country	Case (T/C, n)	Age (T/C, y)	Sex (M/F)	Diagnosis	Chemotherapy	Lentinan	Follow‐up
Cai et al. 11	China	30/34	59 (31–82)	47/17	Advanced lung cancer	DNR + ADM + CTX + DDP	2 mg, twice a week	8 weeks
Ina et al. 12	Japan	31/37	67/68	48/20	Metastatic/recurrent GC	Paclitaxel + S−1 + DDP	2 mg, twice a month	6 years
Guo et al. 13	China	20/20	56 (23–75)	23/17	Lung cancer + GC	5‐FU + ADM + MMC + DNR + DDP	1 mg, twice a week	8 weeks
Ma et al. 14	China	43/42	62.6 ± 5.7	40/45	Advanced GI cancer	Javanica oil emulsion	1 mg, twice a week	8 weeks
Yoshino et al. 15	Japan	149/146	73/74	208/87	Unresectable/recurrent GC	S‐1	2 mg, once a week	12 months
Nakano et al. 16	Japan	23/22	63/66	34/11	Unresectable/recurrent GC	Tegafur + DDP	2 mg, every day	22 months
Ochiai et al. 17	Japan	45/44	60/63	62/27	Advanced/recurrent GC	Tegafur + MMC	2 mg, once a week	>2 years
Suto et al. 18	Japan	15/16	62/66	21/10	HCC	5‐FU	2 mg, once a week	>3 years
Taguchi et al. 19	Japan	111/104	61/59	151/64	Advanced/recurrent GC	5‐FU + MMC	2 mg, once a week	>4 years
Wu et al. 20	China	40/40	68 (55–80)	49/31	Advanced GC	5‐FU + DDP + docetaxel	1 mg, twice a week	4 weeks
Wang et al. 21	China	25/25	60/61	30/20	Esophagus cancer	Tegafur	1 mg, every 2 days	6 weeks
Wakui et al. 22	Japan	20/22	<80	26/16	Advanced GC and colorectal cancer	Tegafur	2 mg, once a week	>4 years
Yang et al. 23	China	31/24	60/59	41/14	HCC	Epirubicin + HCPT	500 mg, every day	18 months
Li et al. 24	China	40/40	58 (25–70)	48/32	Advanced GC	Paclitaxel + 5‐FU + DDP	2 mg, once a week	6 weeks
Pan et al. 25	China	23/20	53 (21–74)	34/9	Advanced lung cancer + GC	5‐FU + ADM + MMC + CTX + VCR	1 mg, twice a week	3 months
Song et al. 26	China	30/30	65 (60–67)	48/12	Advanced lung cancer	MMC + VCR + DDP	2 mg, twice a week	8 weeks
Wang et al. 27	China	42/39	>18	65/16	Advanced lung cancer	NVP + DDP	1 mg, twice a week	8 weeks

T, treatment group; C, control group; M, male; F, female; GI, gastrointestinal; GC, gastric cancer; HCC, hepatocellular carcinoma; ADM, adriamycin; CTX, cyclophosphamide; DDP, cisplatin; DNR, daunorubicin; 5‐FU, fluorouracil; HCPT, hydroxycamptothecin, MMC, mitomycin C; VCR, vincristine; NVP, vinorelbine.

Chemotherapy agents were different across the included trials. Although all of them were platinum‐, paclitaxel‐ and fluorouracil‐based regimens, five trials adopted one single agent, five trials adopted two combined agents, and seven trials adopted three and more agents. There were also some differences in aspects of lentinan usage and course, except one trial perfused lentinan locally in tumor tissue though transcatheter hepatic arterial chemoembolisation, the others injected lentinan intravenously. Lentinan were used once a day in two trials, every 2 days in one trial, twice a week in seven trials, once a week in six trials and twice a month in one trial. A single dose of 2 mg were used in 10 trials and 1 mg in seven trials. For outcome measurements, nine trials had a follow‐up period less than 4 months, three trials had a follow‐up between 1 and 2 years, and five trials had a follow‐up more than 2 years.

**Figure 2:**
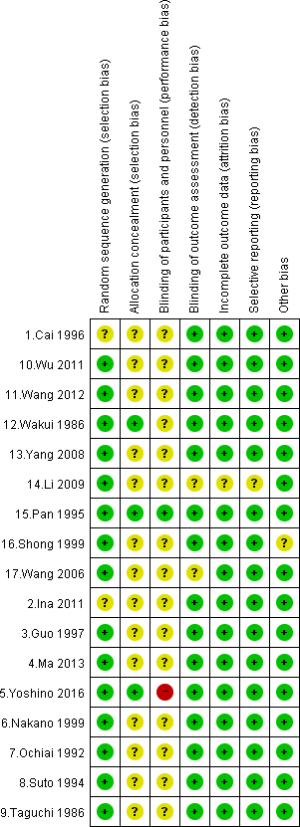
Summary of risk of bias.

Figure 2 shows the risk of bias assessment results, and it was supposed that the overall quality would be moderate as a majority of them did not report sufficient information on allocation concealment and blinding of participants. For four trials judged as low risk for the former item, a center controlled system or a sealed envelop method was adopted 15, 18, 22, 25; one trial judged as low risk for the later item used a placebo similar to lentinan 25.

### Survival rate

**Figure 3:**
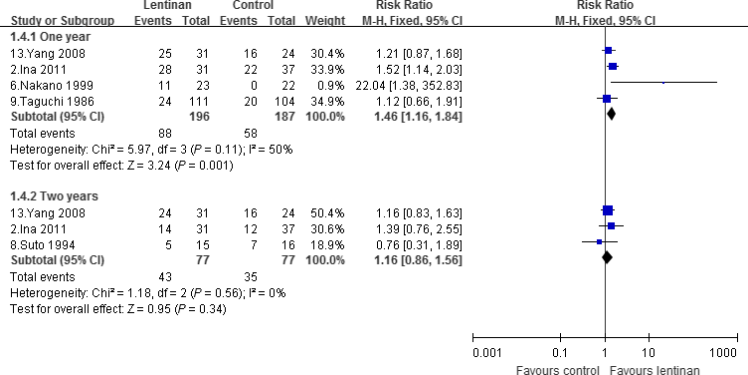
Survival rate.

The pooled survival rate were 44.89% in the lentinan group and 31.02% in the control group in 1 year 2, 6, 9, 13, and 55.84/45.45% in 2 years 2, 8, 13. There was s significant increase of survival rate in lentinan group compared with control in 1 year [RR, 1.46, 95% CI (1.16–1.84); *P *=* *0.001], whereas no significant difference was found in 2 years [1.16 (0.85–1.56); *P *=* *0.34]; as shown in Figure 3. There was a modest amount heterogeneity in 1‐year point (*I* ^2^ = 50%). Sensitivity analysis excluding the study of Nakano et al. 16 indicated that heterogeneity was apparently lower (*I* ^2^ = 0%), whereas the trend of survival rate was maintained [1.29 (1.02, 1.61); *P *=* *0.03].

### Survival time

Four trials reported the data of median overall survival 12, 13, 15, 18. They were not synthesized because a statistical heterogeneity was presented (*I* ^2^ = 77%) and a large‐scale study 15 would significantly affect the overall estimates. Descriptive analysis was chosen. Two trials containing GC patients reported different results, as the large‐scale one 15 including 295 cases was negative [median, 9.90, 95% CI (7.90–12.0) vs. 13.8 (11.80–15.80) months; *P *=* *0.21], whereas the other one 12 including 68 cases was positive [median, 689, (431–2339) vs. 565 (323–662) days; *P *=* *0.04]. One trial 18 including 31 HCC patients reported no significant difference between the groups [389.80 ± 70 vs. 742.50 ± 123.20 days; n.s.], whereas the other one 13 including 55 HCC patients reported a significant longer survival in favor of lentinan [28.20 vs. 21.90, *P *<* *0.05].

### Time to treatment failure

Two trials reported the data 15, 18, however, significant differences were located in both their definitions and results. The study of Yoshino et al. 15 analyzed all possible causes of treatment discontinuation including disease progression, treatment toxicity and patients’ or doctors’ decision, and showed that lentinan was associated with a worse results compared with control [median, 2.60, 95% CI (2.20–3.00) vs. 4.3 (3.80–4.70) months; *P *<* *0.001]. Suto et al. 18 only analyzed patients of disease progression and found no significant difference between lentinan and control [344.10 ± 266.30 vs. 299.30 ± 209.50; n.s.].

### Overall response rate

**Figure 4:**
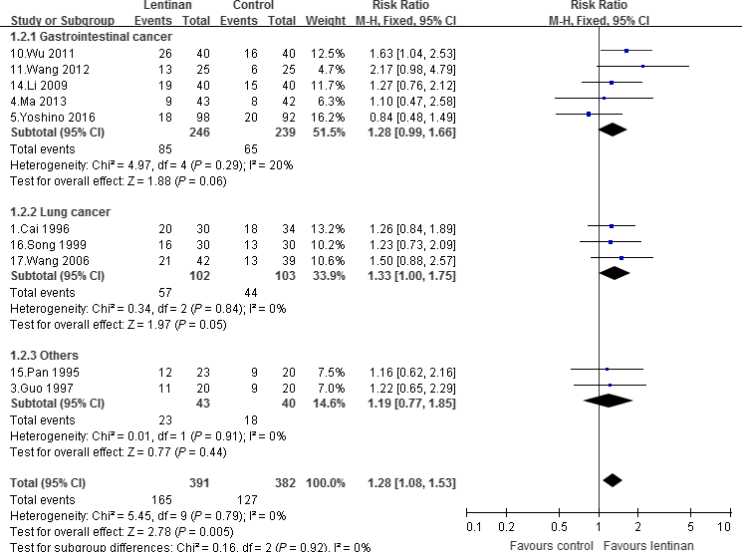
Overall response rate.

The pooled overall response rate of 10 trials was 42.20% in the lentinan group and 33.25% in the control group as shown in Figure 4 1, 3, 4, 5, 10, 11, 14, 15, 16, 17. There was a significant increase in overall response rate in lentinan group than that seen in control group [RR, 1.28, 95% CI (1.08–1.55); *P *=* *0.005]. Stratifying according to the type of cancer, no significant difference was found in both subgroups for GIC and others, whereas there was a boundary combined estimate in the LC subgroup [1.33 (1.00–1.75); *P *=* *0.05].

### Complete response and partial response

**Figure 5:**
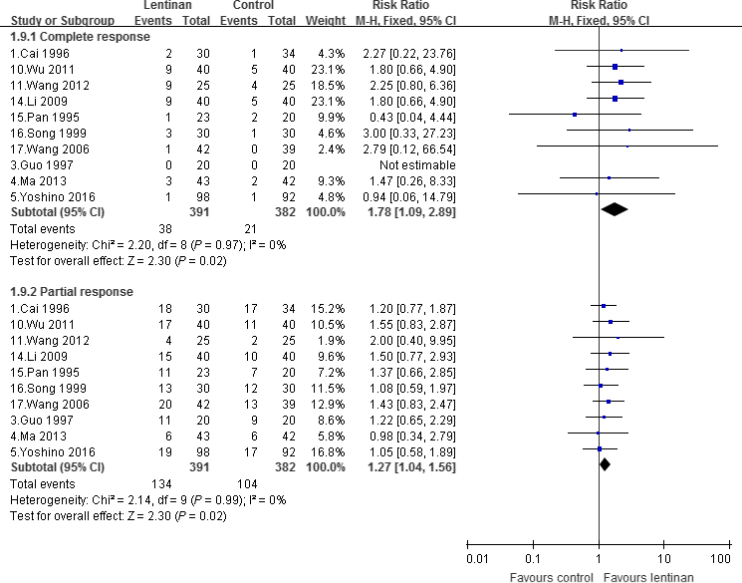
Complete response and partial response.

Overall complete response rate was 9.19% in lentinan group and 5.65% in control group 1, 3, 4, 5, 10, 11, 14, 15, 16, 17, and partial response rate was 36.22% and 29.84% 1, 3, 4, 5, 10, 11, 14, 15, 16, 17, respectively. There was a significant increase in complete response [RR, 1.78, 95% CI (1.09–2.89); *P *=* *0.02] and a significant increase in partial response [1.27 (1.04–1.56); *P *=* *0.02]; as shown in Figure 5. Stratifying according to the type of cancer, only subgroup containing GIC patients showed a significant increase in complete response [1.81 (1.06–3.12); *P *=* *0.03], whereas no significant difference was found in other subgroups of LC and others in both complete response and partial response.

### Progressive disease

A total of nine trials reported the data of progressive disease 1, 3, 4, 11, 14, 15, 16, 17. The pooled rate was 14.49% and 25.36% in lentinan group and control group, respectively. There was a significant reduction associated with lentinan compared with control [RR, 0.57, 95% CI (0.41–0.78); *P *=* *0.0005]. Subgroup of both GIC patients [0.59 (0.41–0.87); *P *=* *0.005] and LC patients [0.34 (0.16–0.72); *P *=* *0.007] showed significant reductions compared with control.

### Adverse events

The reported adverse events were separated into nonsevere subgroup with a grade of 1 or 2 and severe subgroup with a grade of 3 or 4. Nonsevere adverse events were significantly lower in the lentinan group than that in the control group [RR, 0.88, 95% CI (0.81–0.96); *P *=* *0.004], and so were severe adverse events [0.73 (0.58–0.92); *P *=* *0.007]. Stratifying according to the type of diagnosis, except for a significant reduction in nonsevere thrombocytopenia [0.77 (0.61, 0.98); *P *=* *0.03] and severe gastrointestinal reactions [0.91 (0.50–1.65); *P *=* *0.0009] in the lentinan group, no significant difference was found between lentinan and control in aspects of neutropenia, mucositis, anemia, and infections; as shown in Table 2.

**Table 2:** Summary of adverse events in the meta‐analysis

	Neutropenia	Mucositis	Thrombocytopenia	Anemia	Gastrointestinal reaction	Infection	Overall
Nonsevere	0.84 (0.69, 1.02) *I* ^2^ = 28%, *P *=* *0.09	0.98 (0.50, 1.93) *I* ^2^ = 0, *P *=* *0.95	0.77 (0.61, 0.98) *I* ^2^ = 46%, *P* = 0.03^a^	0.91 (0.77, 1.09) *I* ^2^ = 34%, *P* = 0.31	0.93 (0.81, 1.06) *I* ^2^ = 17%, *P *=* *0.27	0.92 (0.46, 1.84) *I* ^2^ = 19%, *P* = 0.82	0.88 (0.81, 0.96) *I* ^2^ = 13%, *P* = 0.004^a^
Severe	0.80 (0.56, 1.13) *I* ^2^ = 2%, *P* = 0.20	1.13 (0.50, 2.54) *I* ^2^ = 0, *P* = 0.77	1.10 (0.39, 3.12) *I* ^2^ = 59%, *P* = 0.85	0.91 (0.50, 1.65) *I* ^2^ = 0, *P* = 0.76	0.46 (0.29, 0.73) *I* ^2^ = 0, *P* = 0.0009^a^	0.47 (0.11, 2.04) *I* ^2^ = 0, *P* = 0.32	0.73 (0.58, 0.92) *I* ^2^ = 1%, *P* = 0.007^a^

a *P* value less than 0.05.

### Publication bias and TSA

**Figure 6:**
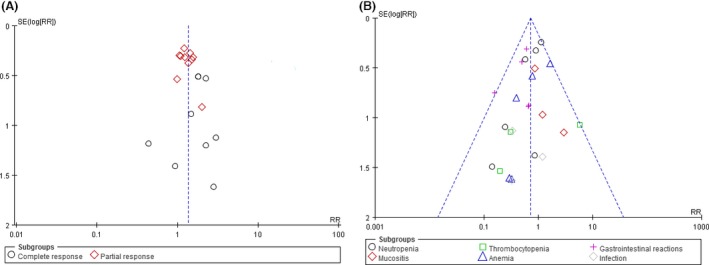
Inverted funnel plots (A, response rate; B, severe adverse events).

**Figure 7:**
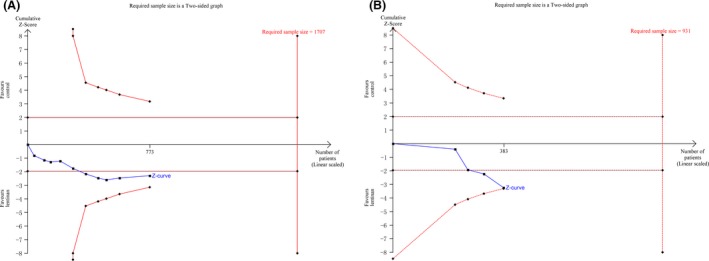
Trial sequence analysis (A, partial response rate; B, 1 year survival rate).

By visually judging the asymmetry and the results of Egger's and begg's tests, low risk of publication bias may be associated with survival rate in 1 year (Egger: *P *=* *0.396; Begg: *P *=* *0.734), overall response (*P *=* *0.941; *P *=* *0.474), complete response (*P *=* *0.461; *P *=* *0.677) and partial response (*P *=* *0.521; *P *=* *0.371), progressive disease (*P *=* *0.395; *P *=* *0.711), nonsevere (*P *=* *0.190; *P *=* *0.114), and severe adverse events (*P *=* *0.130; *P *=* *0.442); as shown in Figure 6. TSA for two positive outcome measures of survival rate in 1 year and partial response showed that they are both under the required sample size when type I error set as 5% and type II error set as 20% in a two‐side test, with an estimated increase in 13.87% and 6.38% from this combined analysis; but, it revealed that survival rate was associated with very low risk of false positive as *Z*‐curve crossed both of the other two boundary lines; as shown in Figure 7.

## Discussion

In this critical meta‐analysis, we have systematically reviewed current evidence of adjuvant lentinan combined with chemotherapy for kinds of cancers including GIC and LC. The study was designed to comprehensively determine the clinical therapeutic effects of lentinan by multiple outcome measures. We found that there was a significant increase in survival rate in 1‐year follow‐up, and also there were significant improvements of short‐term evaluation in aspects of objective response and progressive disease. Besides, additional lentinan was associated with lower incidence of adverse events compared with chemotherapy alone.

As lentinan is currently considered to be one of nonspecific BRMs, its immune modifying effects would be beneficial to various kinds of cancers 2, 3. For advanced cancer, host immune impair are common; besides, burdens from depression, surgery, and chemotherapy agents may also aggravate the suppressed situation of immune response 28. Previous studies mentioned that lentinan may induce a upregulation of T helper 1 (Th1) response and a downregulation of Th2 response, which was helpful to adjust the Th1/Th2 imbalance 29. Although shifting imbalance from Th2 dominated to Th1, cellular immunity seemed to be enhanced to contribute to potential clinical benefits. Besides, lentinan is supposed to have a possibility of direct anticancer or sensitizing effects on specific chemotherapy agent. Recommended chemotherapy regimens were different for GIC and LC. Across the included studies, fluorinated pyrimidines‐based drugs were mostly used for GIC but not for LC. The combination of fluorinated pyrimidines and lentinan was described previously 5, and also combination of S‐1 and lentinan was also explored 15, however, different effects of additional lentinan were presented. Some other studies also showed that in vitro, inhibition effects can be significantly enhanced when they were combined with monoclonal antibodies and gemcitabine 30, 31. Therefore, combined effects on the whole population and separate effects on GIC and LC were both considered and evaluated in this study.

For the whole population composed of 1423 cancer patients, adjuvant lentinan achieved an increase of 1‐year survival rate, both sensitivity analysis and TSA analysis demonstrated its stability. Survival time as well as time to treatment failure was also important endpoints in anticancer research, whereas they were not combined in our analysis. A large heterogeneity was existed in both of the two outcomes, and also limited number of studies reported the data. Under such a situation, quantitative analysis results would be severely influenced by some large scale or estimate studies, so we only performed a descriptive analysis and obviously the results were still not clear. There was a significant reduction in complete response rate and partial response rate, and a significant increase in progressive disease rate. Compared with primary outcome of long‐term survival, these secondary outcomes could be easily measured in relative short time, however, incapable to exactly reflect overall or sustained treatment effects of chemo‐immunotherapy with lentinan. For separate effect on GIC and LC, lentinan was combined with different chemotherapy regimens, and no significant difference was found between the subgroup of GIC and LC as well as corresponding chemotherapy agents. Thus lentinan may act mainly as one kind of BRMs other than a specific sensitizer for some chemotherapy agents adopted in current analysis for GIC and LC.

The safety of lentinan are certain, because it comes from medical mushroom as well as traditional food in Asian, and is also proven by randomized trials in healthy elderly 32, 33. Current study showed a possible effects of lentinan on declining the incidence of chemotherapy‐related adverse events. Further analysis identified that gastrointestinal reactions and thrombocytopenia were significant reduced. Retrospective study of Higashi et al. reported similar results and found a longer duration of chemotherapy for patients taking lentinan, and guessed that less‐frequency adverse events may contribute to tolerance of chemotherapy and improvement of quality of life 6. One randomized trial focused on quality of life in women with breast cancer undergoing chemotherapy using the Global health status/QoL score in 3 weeks reported positive results 34, whereas the included study by Yoshino et al. did not find any difference on changes in quality of life at posttreatment 4, 6, 10, and 12 weeks though a self‐administered and not obligatory scale 15. As quality of life assessment for elderly patients is becoming more and more important in the comprehensive treatment of cancer except for survival, future study focused this issue are warranted although different kinds of assessment scale may indicate varied results.

Many studies discussed the mechanism of polysaccharide on its various bioactivities. Among them, beta glucan is one large part, and in this part the effects and mechanisms are probably determined by the branching and/or polymer length in the structure 4. Lentinan is one specific beta glucan widely used as medicine in clinic through parental injection and also sometimes as nutritional products by orally taking. For healthy elderly, it was found to increase B‐cell count and the level of IFN‐*γ* after administration, whereas NK cell and other cytokine including IL‐2, IL‐4, IL‐10, IL‐12, and TNF‐*α* were not changed 32, 33. More complex regulatory pathways seemed to be existed for advanced cancer patients. Yoshino et al. reported a maintained or decreased granulocytes/lymphocytes ratio during follow‐up and supposed lentinan may increase the cellular response. Findings from latest basic cell and animal researches of lentinan in vitro and in vivo primary elucidated direct anticancer effects and immune modifying effects: (1) lentinan alone inhibits cancer cell proliferation and induce cell apoptosis 30, 35; (2) a combination of lentinan with chemotherapy drugs or monoclonal antibodies enhances their toxicity to cancer cell 30, 35, 36; (3) lentinan can be blinding to activate macrophages and other monocytes 15. Anticancer effects were found to be mainly p53‐dependent, and involved MAPK signaling pathways activation and reactive oxygen species overproduction 37, 38; and immune modifying effects of lentinan mainly because of its activation of macrophages and monocytes though binding to specific receptors on the cells, and further induction of cytokine release, complement activation, and antibody‐dependent cell‐mediated cytotoxicity 4.

Limitations except for methodological quality of the included studies in the meta‐analysis should be mentioned. The effects of lentinan on survival time and treatment to failure were unclear, and clinical data and sample size were both insufficient to conduct this analysis. Although amount of basic researches pushed forward our understanding of lentinan, there was still far away to draw a conclusion on the issues. Differences located in dose, course, and manufacturer of lentinan inevitably induced a heterogeneity. Optimal administration method was not recommended in any of the papers and was also unclear although basic study showed a significant dose‐dependent manner of anticancer effect 30. In the included studies from China and Japan it seemed to have verified dose and course for kinds of cancers, and also two nonrandomized prospective studies were included, after all the heterogeneity were mostly small and the results were stable. Besides, all the studies focused this agents purified from traditional medicine were conducted in China and Japan, thus region bias or publication bias may be also existed.

In conclusion, adjuvant lentinan as one of BRMs combined with chemotherapy led to clinical improvements for advanced cancer patients in aspects of 1‐year survival, objective response and chemotherapy‐related adverse events. However, its long‐term efficacy on overall survival warranted more large‐scale studies.

## Ethical Approval

This article does not contain any studies with human participants or animals performed by any of the authors.

## Conflict of Interest

All the authors declare no conflicting interests.
